# The Fungal Pathogen *Candida glabrata* Does Not Depend on Surface Ferric Reductases for Iron Acquisition

**DOI:** 10.3389/fmicb.2017.01055

**Published:** 2017-06-08

**Authors:** Franziska Gerwien, Abu Safyan, Stephanie Wisgott, Sascha Brunke, Lydia Kasper, Bernhard Hube

**Affiliations:** ^1^Department of Microbial Pathogenicity Mechanisms, Leibniz Institute for Natural Product Research and Infection Biology – Hans Knoell InstituteJena, Germany; ^2^Department of Microbial Pathogenicity Mechanisms, Friedrich Schiller UniversityJena, Germany; ^3^Center for Sepsis Control and Care, University HospitalJena, Germany

**Keywords:** *Candida glabrata*, iron, ferric reductase, fungi, extracellular ferric reduction

## Abstract

Iron acquisition is a crucial virulence determinant for many bacteria and fungi, including the opportunistic fungal pathogens *Candida albicans* and *C. glabrata.* While the diverse strategies used by *C. albicans* for obtaining iron from the host are well-described, much less is known about the acquisition of this micronutrient from host sources by *C. glabrata* – a distant relative of *C. albicans* with closer evolutionary ties to *Saccharomyces cerevisiae*, which nonetheless causes severe clinical symptoms in humans. Here we show that *C. glabrata* is much more restricted than *C. albicans* in using host iron sources, lacking, for example, the ability to grow on transferrin and hemin/hemoglobin. Instead, *C. glabrata* is able to use ferritin and non-protein-bound iron (FeCl_3_) as iron sources in a pH-dependent manner. As in other fungal pathogens, iron-dependent growth requires the reductive high affinity (HA) iron uptake system. Typically highly conserved, this uptake mechanism normally relies on initial ferric reduction by cell-surface ferric reductases. The *C. glabrata* genome contains only three such putative ferric reductases, which were found to be dispensable for iron-dependent growth. In addition and in contrast to *C. albicans* and *S. cerevisiae*, we also detected no surface ferric reductase activity in *C. glabrata*. Instead, extracellular ferric reduction was found in this and the two other fungal species, which was largely dependent on an excreted low-molecular weight, non-protein ferric reductant. We therefore propose an iron acquisition strategy of *C. glabrata* which differs from other pathogenic fungi, such as *C. albicans*, in that it depends on a limited set of host iron sources and that it lacks the need for surface ferric reductases. Extracellular ferric reduction by a secreted molecule possibly compensates for the loss of surface ferric reductase activity in the HA iron uptake system.

## Introduction

Iron is an essential micronutrient for almost all living organisms (Posey and Gherardini, 2000; Troxell et al., 2012), as it is indispensable for numerous cellular processes such as respiration, synthesis of iron-sulfur-clusters (Fe-S clusters), the tricarboxylic acid (TCA) cycle, and the synthesis of DNA, amino acids, lipids, and sterols (Schaible and Kaufmann, 2004). Although iron is highly abundant in the environment, its bioavailability is low due to the low solubility of its most common form, ferric iron (Fe^3+^), under aerobic conditions (Haas et al., 2008). For pathogenic microbes, iron acquisition is especially demanding, since they are dependent on the host’s iron supply. However, host iron is typically bound to carrier proteins such as hemoglobin, the transport compound transferrin, or the storage molecule ferritin (Ratledge, 2007). Additionally, iron is also actively withheld from infection sites to restrict the proliferation of invading pathogens. This host strategy is known as nutritional immunity (Hood and Skaar, 2012).

Successful pathogens, including many fungal species, have therefore evolved sophisticated strategies to use iron sources in the host, and these strategies are considered important virulence attributes (Timmerman and Woods, 1999; Almeida et al., 2008; Jung et al., 2008; Newman and Smulian, 2013; Kuznets et al., 2014). The opportunistic fungal pathogen *Candida albicans* and its distant relative *C. glabrata*, for example, are members of the normal gastrointestinal and oral flora, but can also cause diseases ranging from superficial mucosal infections to life-threatening systemic infections (e.g., candidemia), especially in immuno-compromised hosts (Perlroth et al., 2007). *C. albicans* is the most frequently encountered species causing candidemia, while *C. glabrata* ranks second in Europe and the United States (Guinea, 2014). However, despite colonizing the same host niche and causing similar clinical symptoms, *C. glabrata* is evolutionarily more closely related to the baker’s yeast *Saccharomyces cerevisiae* than to *C. albicans.* In fact, *C. glabrata* and *C. albicans* differ remarkably from each other regarding their infection strategy, morphological flexibility, and genome structure, as *C. glabrata* possesses a haploid genome, and lacks certain virulence-associated genes and metabolic pathways known in other yeasts (Brunke and Hube, 2013). As recently shown, this species difference also extends to the regulatory networks for iron homeostasis (Blankenship and Mitchell, 2011; Chen et al., 2011; Gerwien et al., 2016).

Most pathogenic fungi have three main iron acquisition strategies in common: (i) the receptor-mediated uptake of siderophores (excreted low-molecular weight, high-affinity iron scavengers); (ii) the receptor-mediated heme uptake; and (iii) the reductive high-affinity (HA) iron uptake. Although *Candida* and *Saccharomyces* species do not produce their own siderophores, they are generally able to use siderophores produced by other microorganisms (xenosiderophores) (Haas et al., 2008; Nevitt and Thiele, 2011). In *C. albicans*, heme/hemoglobin is additionally bound by receptors of the Rbt family (predominantly Rbt5 and Rbt51), followed by endocytosis and intracellular degradation by a heme oxygenase (Weissman and Kornitzer, 2004; Weissman et al., 2008).

The reductive HA iron uptake is strictly required for virulence in *C. albicans* (Ramanan and Wang, 2000; Almeida et al., 2008; Cheng et al., 2013) and is not only crucial for uptake of free iron, but also for acquisition of iron associated with transferrin (Knight et al., 2005) or ferritin (Almeida et al., 2008). Typically, the reductive HA iron uptake consists of three steps: (i) initial extracellular Fe^3+^ reduction to Fe^2+^, typically mediated by surface-bound ferric reductases followed by (ii) re-oxidation to Fe^3+^ by multicopper ferroxidases, and (iii) Fe^3+^ import by the permease Ftr1. Ferric reduction is therefore important for releasing complexed ferric iron (bound, e.g., to siderophores, transferrin, or ferritin) into the soluble ferrous state (Schröder et al., 2003). In addition to iron uptake, ferric reductases play a central role in intracellular iron transmembrane transport and storage, when present in the vacuolar membrane (Urbanowski and Piper, 1999; Haas et al., 2008), and in some cases they serve as surface cupric reductases (Martins et al., 1998; Woodacre et al., 2008; Jeeves et al., 2011). Consequently, many fungi have large families of ferric reductases with diverse functions encoded in their genome, among them *S. cerevisiae* (nine characterized ferric reductases) (Dancis et al., 1992; Martins et al., 1998; Yun et al., 2001) and *C. albicans* (17 putative ferric reductases) (Jeeves et al., 2011; Xu et al., 2014). In contrast, the ferric reductases of *C. glabrata* are not well characterized yet, and our knowledge of this fungus’ mechanisms for exploiting host iron sources is still incomplete. The aim of this study was therefore to investigate the iron acquisition strategies of *C. glabrata*, and in particular its spectrum of host iron sources and the nature of the ferric reduction in its high-affinity iron uptake system.

We report here that *C. glabrata* can use non-protein-bound iron (FeCl_3_) and ferritin-associated iron, but not iron bound to transferrin, hemin, or hemoglobin, in a pH-dependent manner via the reductive HA iron uptake system. Surprisingly, the two analyzed putative ferric reductases encoded in the *C. glabrata* genome proved to be dispensable for growth, and *C. glabrata* lacks surface ferric reductase activity even under iron starvation. Instead, we found that this fungus produces an extracellular low-molecular, non-protein ferric reductant.

## Results

### Comparative Analysis of Iron Acquisition and Trafficking Systems in *C. glabrata*, *S. cerevisiae*, and *C. albicans*

Fungi use various, partially redundant iron acquisition systems, depending on their current host niche. To find common key components of iron acquisition and trafficking in *C. glabrata*, *S. cerevisiae*, and *C. albicans*, we searched their genome databases (*Candida* Genome Database, CGD, and *Saccharomyces* Genome Database, SGD) for orthologs with known or postulated iron-related functions based on previously published data or iron-related mutant phenotypes (**Table 1** and Supplementary Table 1). Genes involved in intracellular iron trafficking were found highly conserved between the species (Supplementary Table 1). These code for transporters in membranes of the vacuole, a major iron storage site (Ccc1, Smf1, Smf3, and Fth1), and of the mitochondria, where a constant supply of iron is needed to maintain respiration and iron-sulfur cluster biosynthesis (Mmt1, Atm1, Mrs3, and Mrs4). Similarly, all three species possess the main components of the reductive HA iron uptake (the permease Ftr1, the associated ferroxidases Fet3/Fet34, and ferric reductases; **Table 1**). Notably, the ferric reductase family is considerably smaller in *C. glabrata* (3 Fre orthologs) than in *C. albicans* (17 Fre orthologs) and *S. cerevisiae* (9 Fre orthologs). With regard to host iron source-related acquisition systems, however, major differences between the species were observed (**Table 1**). While all three species encode orthologs of transporters for xenosiderophore uptake (Sit1/Arn1-4), both *C. glabrata* and *S. cerevisiae* lack orthologs of *C. albicans* proteins associated with iron acquisition from host ferritin (Als3) or heme and hemoglobin (Rbt5, Rbt51, Csa1, Csa2, and Pga7).

**Table 1:** Iron acquisition systems in *Candida glabrata*, *Saccharomyces cerevisiae* and *Candida albicans.*

Process	Function	*C. glabrata*	*S. cerevisiae*	*C. albicans*
Xenosiderophore uptake	Transporter	Sit1 (Nevitt and Thiele, 2011; Gerwien et al., 2016)	Arn1-4 (Heymann et al., 2000;Yun et al., 2000a;Yun et al., 2000b)	Sit1 (Heymann et al., 2002; Lesuisse et al., 2002)
	Siderophore-binding cell wall mannoproteins	n.o.	Fit1-3 (Protchenko et al., 2001; Philpott et al., 2002)	n.o.

Ferritin uptake	Receptor	n.o.	n.o.	Als3 (Almeida et al., 2008)

Heme/hemoglobin uptake	Receptor	n.o.	n.o.	Rbt5 (Weissman and Kornitzer, 2004)
	Receptor	n.o.	n.o.	Rbt51 (Weissman and Kornitzer, 2004)
	Receptor	n.o.	n.o.	Csa1 (Almeida et al., 2008; Singh et al., 2011)
	Receptor	n.o.	n.o.	Csa2 (Okamoto-Shibayama et al., 2014)
	Cell wall protein	n.o.	n.o.	Pga7 (Kuznets et al., 2014)
	Heme oxygenase	Hmx1 (Gerwien et al., 2016)	Hmx1 (Protchenko and Philpott, 2003; Kim et al., 2006)	Hmx1 (Santos et al., 2003)

Reductive HA iron uptake	HA Fe^3+^ transporter complex	Ftr1/Fet3 (Srivastava et al., 2014; Gerwien et al., 2016)	Ftr1/Fet3 (De Silva et al., 1995; Stearman et al., 1996)	Ftr1/Fet34 (Ramanan and Wang, 2000; Ziegler et al., 2011)
	HA Fe^3+^ transporter	n.o.	n.o.	Ftr2**^∗^**(Ramanan and Wang, 2000)
	Ferric reductases Fe^3+^ → Fe^2+^	3 × Fre^#^ (Srivastava et al., 2014)	9 × Fre (Martins et al., 1998; Yun et al., 2001)	17 × Fre^+^ (Baek et al., 2008; Jeeves et al., 2011; Xu et al., 2014)
	Multicopper ferroxidases Fe^2+^ → Fe^3+^	5 × Fet^#^ (Srivastava et al., 2014)	3 × Fet (Spizzo et al., 1997)	5 × Fet (Ziegler et al., 2011; Cheng et al., 2013)

LA metal uptake	Divalent metal transporter (including Fe^2+^)	Fet4 (Gerwien et al., 2016)	Fet4 (Hassett et al., 2000; Jensen and Culotta, 2002)	n.o.**^∗^**

*No ortholog (n.o.), High affinity (HA), Low affinity (LA). ^#^Not all orthologs are characterized. ^+^Orf19.1845, which was additionally predicted as an 18th member of the Fre family (Jeeves et al., 2011), was merged with CaFre4 in Assembly 20. ^∗^Ca*FTR2* expression has been shown to be induced under iron exposure, indicating a function resembling a LA-transporter or an intracellular HA Fe^3+^ permease (Knight et al., 2002)*.

Thus, although *C. glabrata* is a successful pathogen with a large overlap in host niches to *C. albicans*, it seems to lack the components needed for the utilization of the most abundant host iron sources.

### Use of Host Iron Sources Is Restricted to Non-protein-bound Iron and Ferritin in *C. glabrata*

**FIGURE 1:**
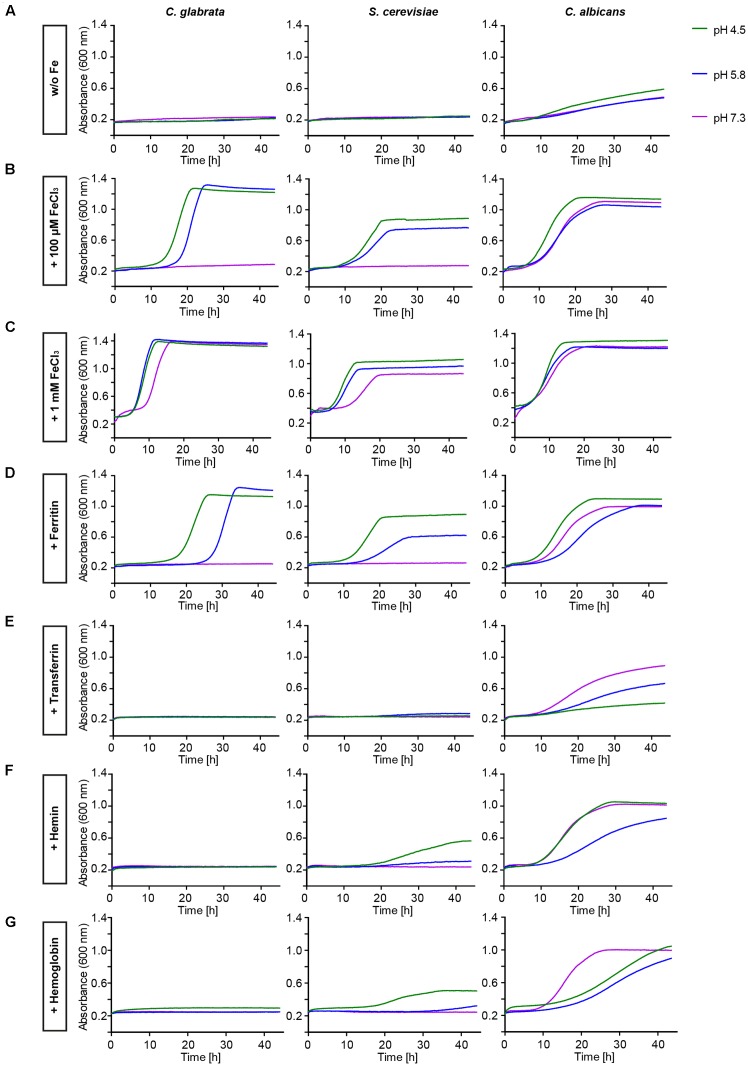
Comparative pH-dependent growth of *Candida glabrata*, *Saccharomyces cerevisiae*, and *Candida albicans* on host iron sources. Growth of iron-prestarved [preculture treatment with the iron chelator bathophenanthrolinedisulfonic acid (BPS)] wild-type (WT) strains in liquid SD buffered to pH 4.5, pH 5.8, and pH 7.3 containing: **(A)** 200 μM BPS (*S. cerevisiae*, *C. glabrata*) or 500 μM BPS (*C. albicans*) **(B)** BPS + 100 μM FeCl_3_ **(C)** BPS + 1 mM FeCl_3_ **(D)** BPS + 100 μg/ml ferritin **(E)** BPS + 100 μg/ml transferrin **(F)** BPS + 1 μM hemin **(G)** BPS + 0.1 mg/ml hemoglobin. *n* = 3, one representative example is shown.

The *in silico* data indicated a limited set of host iron sources available for utilization by *C. glabrata*. We thus tested different iron sources commonly found in the host. To this end we grew *C. glabrata*, *S. cerevisiae*, and *C. albicans* without free iron, but in presence of ferritin, transferrin, hemin, or hemoglobin. Addition of 100 μM or 1 mM FeCl_3_ served as a control for moderate or high iron levels. The uptake of xenosiderophores via *C. glabrata* Sit1 has been described elsewhere (Nevitt and Thiele, 2011). To account for possible effects of the pH on host iron source stability and solubility (Almeida et al., 2008), we performed these tests under conditions of different pH (**Figure 1**). Moderate FeCl_3_ levels and ferritin supported robust growth by all three species under acidic pH conditions (pH 4.5 and 5.8). However, under slightly alkaline conditions (pH 7.3) *C. glabrata* and *S. cerevisiae*, but not *C. albicans*, were unable to grow (**Figures 1B,D**). An increase in iron concentration to high levels, however, allowed growth of all species (**Figure 1C**). In agreement with our *in silico* analysis, *C. glabrata* and *S. cerevisiae* were not able to use hemoglobin, hemin, or transferrin over the whole tested range of media pH. In contrast, hemoglobin, hemin, and transferrin were all exploited as iron sources by *C. albicans* in a pH-dependent manner, in which alkaline pH conditions allowed the most robust growth (**Figures 1E–G**).

Taken together, *C. glabrata* is much less versatile than *C. albicans* in the utilization of host iron sources, and more similar to *S. cerevisiae* in that respect. *C. glabrata* thus seems to rely on ferritin and non-protein-bound ferric iron, in combination with acidic pH, for growth under iron-limiting conditions in the host.

### Ferritin and FeCl_3_ Utilization by *C. glabrata* Depends on the Reductive Iron Uptake System

**FIGURE 2:**
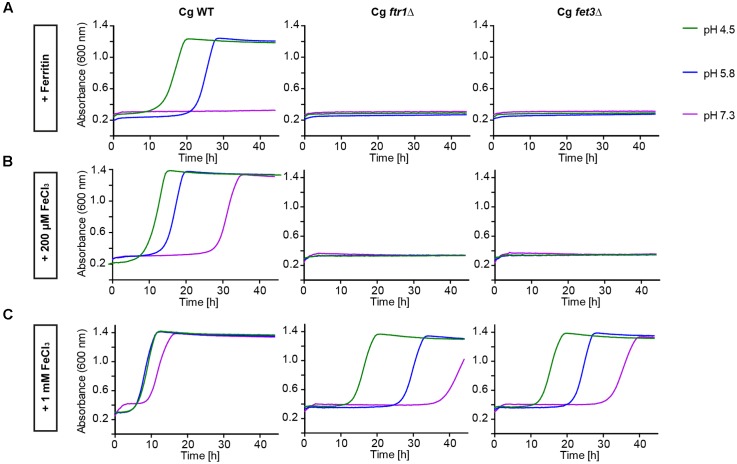
Comparative pH-dependent growth of *C. glabrata* mutants defective in HA reductive iron uptake on host iron sources. Growth of iron-prestarved (preculture treatment with 200 μM BPS) with *C. glabrata* WT, and mutants defective in HA reductive iron uptake (Cg*frt1*Δ and Cg*fet3*Δ) in liquid SD buffered to pH 4.5, pH 5.8, and pH 7.3 at 37°C containing: **(A)** 200 μM BPS + 100 μg/ml ferritin. **(B)** 200 μM BPS + 200 μM FeCl_3._ **(C)** 200 μM BPS + 1 mM FeCl_3_. *n* = 3, A representative example is shown.

To determine which pathways allow *C. glabrata* iron acquisition and growth with ferritin or FeCl_3_ as iron sources, we investigated deletion mutants lacking genes for two major components of the HA iron uptake system – the HA permease Ftr1 and the associated ferroxidase Fet3. Both mutants displayed severe growth defects with either ferritin as sole iron source or in presence of only moderate FeCl_3_ levels. Only high-level iron supplementation (1 mM FeCl_3_) partially restored growth of these mutants, predominantly at acidic pH (**Figure 2**). Hence, the reductive HA iron uptake, particularly the permease-ferroxidase complex, is needed for ferritin- and FeCl_3_-dependent growth of *C. glabrata.*

### Fre Families Differ between *C. glabrata*, *C. albicans*, and *S. cerevisiae*

**FIGURE 3:**
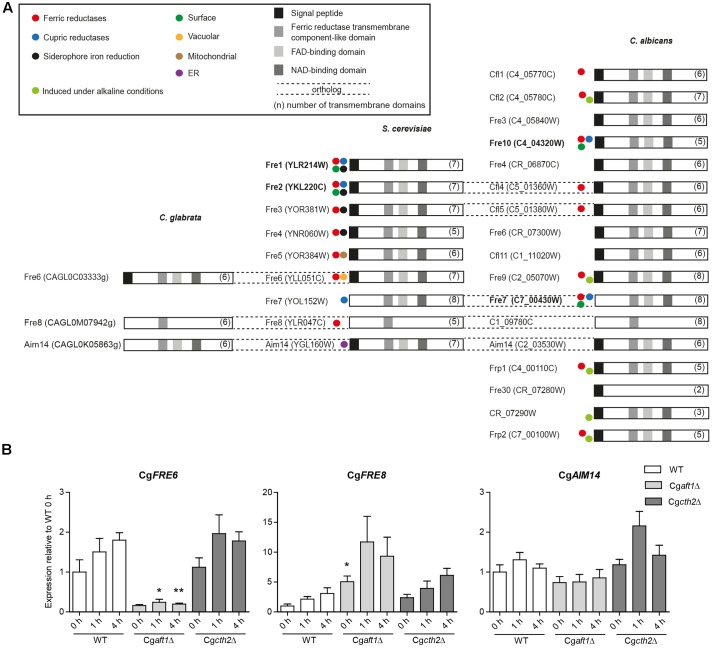
Ferric reductase genes in *C. glabrata*, *S. cereviae* and *C. albicans* and expression under iron deprivation in *C. glabrata*. **(A)** Species comparative *in silico* analysis of the ferric reductase families in *C. glabrata* (3 putative family members), *S. cerevisiae* (9 family members) and *C. albicans* (17 putative family members), Orf19.1845 which had additionally been predicted as a 18th member of the Fre family (Jeeves et al., 2011) was merged with CaFre4 in Assembly 20. Dashed lines = gene orthologs in the respective species. Displayed domains (squares) include the signal peptide, a ferric reductase transmembrane component-like domain (PF01794), a FAD-binding domain (PF08022) and a NAD-binding domain (PF08030). The number of transmembrane domains is indicated in parentheses. Colored circles indicate known activity as ferric, cupric or siderophore iron reductases and the postulated localization on the cell surface, in the vacuole, mitochondria or ER according to previous publications (Supplementary Table 2). Major surface ferric reductases are depicted in bold. **(B)** qRT-PCR expression analysis of the putative *C. glabrata* ferric reductase genes: Cg*FRE6*, Cg*FRE8*, and Cg*AIM14* under iron starvation conditions in WT, Cg*aft1*Δ and Cg*cth2*Δ. Data is shown in biological triplicates as means ± SEM, for statistical analysis unpaired Student’s *t*-test was performed in comparison to WT/equal time point (^∗^*P* < 0.05, ^∗∗^*P* < 0.01, ^∗∗∗^*P* < 0.001).

The initial step of HA iron uptake, the extracellular reduction of ferric iron, is typically mediated by membrane-integral NAD(P)H-dependent ferric reductases (Fre). The *C. glabrata* genome, in contrast to *C. albicans* and *S. cerevisiae* contains only few (three) potential *FRE* gene orthologs (**Table 1**), none of them with proven Fre function. We scanned the genomes of *C. glabrata*, *C. albicans*, and *S. cerevisiae* for additional putative ferric reductases. This *in silico* analysis included a BLAST search for the ferric reductase transmembrane component-like domain (Pfam family PF01794). We identified 17 of the 18 known *C. albicans* ferric reductases (Jeeves et al., 2011), and the nine ferric reductases already described in *S. cerevisiae*. Both families show only marginal overlap, which points to different evolutionary origins in both species. Notably, CaFre10 and ScFre1/ScFre2 (green dot, **Figure 3A**) are known to account for 75% and 90–98% of the whole cell surface reductase activity, respectively (Dancis et al., 1990; Anderson et al., 1992; Georgatsou and Alexandraki, 1994; Knight et al., 2002, 2005), but are non-orthologous. Overall, the gene families of the two species contain ferric as well as cupric reductases, with different cellular localizations depicted as intracellular (mitochondria, ER, vacuole), or extracellular/surface-associated (**Figure 3A** and Supplementary Table 2). Our analysis furthermore revealed that most (potential) Fres additionally contained a FAD-binding domain, a NAD-binding domain, multiple transmembrane domains and a signal peptide (**Figure 3A**).

A BLAST search in the predicted protein sequences of *C. glabrata* using the ferric reductase transmembrane component-like domain of major *C. albicans* and *S. cerevisiae* surface ferric reductases (CaFre10 and ScFre1) as the query revealed only the three aforementioned putative *C. glabrata* ferric reductases CgFre6, CgFre8, and CgAim14. No additional, thus far unknown ferric reductases were found. CgFre6 showed 37% protein identity to ScFre6 and contained the characteristic ferric reductase domains – an N-terminal signal peptide, the ferric reductase transmembrane component-like domain, a FAD- and NAD-binding domain, and a transmembrane domain (**Figure 3**). CgFre8 showed 41% protein identity to ScFre8 and 29.9% identity to its presumed ortholog in *C. albicans*, C1_09780C. Like its *S. cerevisiae* and *C. albicans* orthologs, CgFre8 lacks both, the FAD-/NAD-binding domains and a signal peptide. CgAim14 showed 38 and 28.3% protein identity to its *S. cerevisiae* and *C. albicans* orthologs, respectively. In contrast to its cross-species counterparts, however, CgAim14 does not contain any signal peptide. Importantly, the closest *S. cerevisiae* and *C. albicans* orthologs of CgFre6, CgFre8, and CgAim14 have all not been described as surface-localized ferric reductases. In addition, and in contrast to CgFre6, the lack of a predicted signal peptide in CgFre8 and CgAim14 suggests that these proteins may not enter the secretory pathway and thus do not have access to the cell surface or the extracellular space.

In conclusion, our *in silico* analysis confirmed the comparatively small number of three ferric reductase family members and the lack of orthologs of known surface ferric reductases of *S. cerevisiae* and *C. albicans* in *C. glabrata*. The presence of ferric reductase protein domains and a signal peptide in CgFre6, which is absent in the other putative *C. glabrata* ferric reductases, suggests CgFre6 to be the potential major surface reductase of this species.

### Expression of *CgFRE6* and *CgFRE8* Is Dependent on the Iron Master Regulator Aft1

Expression of uptake-associated ferric reductases is typically induced under iron deprivation (Georgatsou and Alexandraki, 1999; Jeeves et al., 2011). Similarly to *S. cerevisiae* (Puig et al., 2005), iron acquisition in *C. glabrata* is largely activated by the transcription factor Aft1, whereas iron consuming processes are inhibited by Cth2-dependent degradation of their mRNA transcripts (Gerwien et al., 2016).

To analyze the iron-dependent regulation of the three potential *C. glabrata* ferric reductases, we tested for Aft1 and Cth2 dependency of Cg*FRE6*, Cg*FRE8*, and Cg*AIM14* transcription under iron limitation. We found a moderate induction of Cg*FRE6* and Cg*FRE8* transcript levels in the wild-type (WT), in line with their presumed roles as ferric reductases (**Figure 3B**). Cg*AFT1* deletion almost completely abolished Cg*FRE6* expression, but led to an overexpression of Cg*FRE8*. In contrast, both Cg*FRE6* and Cg*FRE8* were not significantly affected by *CTH2* deletion. Finally, transcript levels of Cg*AIM14* were largely unaffected by iron deprivation and by deletion of Cg*AFT1* or Cg*CTH2*.

Taken together, the CgAft1-dependent suppression indicates that there is no apparent role of CgFre8 in iron uptake, while the CgAft1-mediated up-regulation of Cg*FRE6* transcription supports our *in silico* prediction of CgFre6 as the main *C. glabrata* uptake-associated surface ferric reductase.

### *C. glabrata* Fre6 and Fre8 Do Not Confer Ferric Reductase Activity *In Vitro*

**FIGURE 4:**
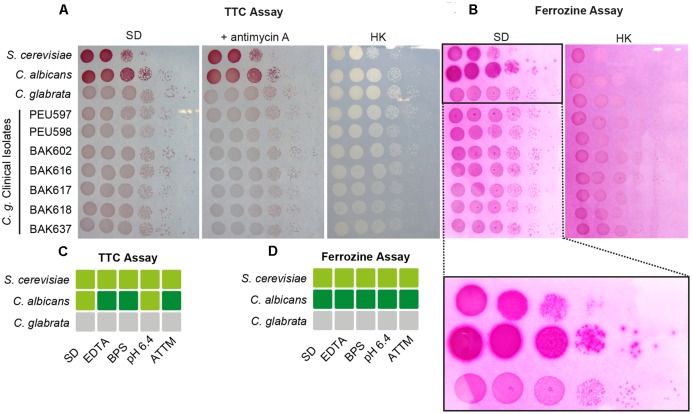
*Candida glabrata* lacks surface ferric reductase activity. Species comparison of surface ferric reductase activity for *S. cerevisiae*, *C. albicans*, and *C. glabrata* WTs as well as *C. glabrata* clinical isolates derived from different host tissues. Cells were spotted on SD agar and grown over-night (oN) at 37°C. **(A)** Determination of surface reductase activity with 2,3,5-triphenyltetrazolium chloride (TTC). Red coloration of colonies 1 h after application indicates surface reductase activity. Antimycin A was added to inhibit intracellular respiratory reduction. HK: heat-inactivated plate (70°C, 1 h). **(B)** Determination of surface ferric reductase activity with ferrozine. Formation of a purple halo (see magnified section) around colonies 5 min after application of ferrozine indicates surface ferric reductase activity. HK: heat-inactivated plate (70°C, 1 h) represents negative control. **(C,D)** Summary of determined surface reductase activity with TTC **(C)** and ferrozine **(D)** under iron- and copper-deprivation inducing conditions (3 μM ethylenediaminetetraacetic acid (EDTA), 10 μM BPS, pH 6.4, 7 μM ammonium tetrathiomolybdate (ATTM), gray box: no surface reductase activity, light-green box: moderate surface reductase activity, dark-green box: strong surface reductase activity.

**FIGURE 5:**
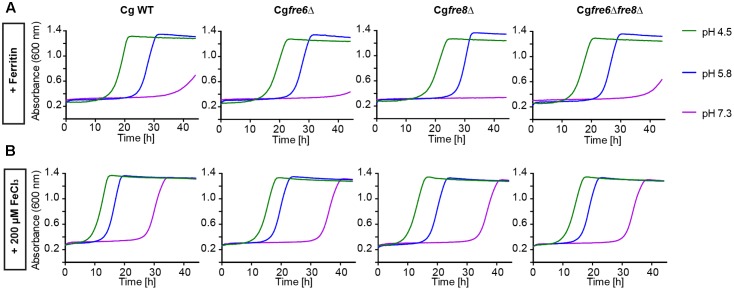
Comparative pH-dependent growth of *C. glabrata* ferric reductase mutants on host iron sources. Growth of iron-prestarved (preculture treatment with 200 μM BPS) with *C. glabrata* WT, and mutants (Cg*fre6*Δ, Cg*fre8*Δ and Cg*fre6*Δ*fre8*Δ) in liquid SD buffered to pH 4.5, pH 5.8, and pH 7.3 at 37°C containing: **(A)** 200 μM BPS + 100 μg/ml ferritin. **(B)** 200 μM BPS + 200 μM FeCl_3_. *n* = 3, A representative example is shown.

We wanted to measure the contribution of these proteins to the surface ferric reductase activity of *C. glabrata* by a 2,3,5-triphenyltetrazolium chloride (TTC)- and ferrozine-based assay (**Figure 4**; see Materials and Methods section). Surprisingly, while *C. albicans* and *S. cerevisiae* exhibited surface ferric reductase activity, indicated by red coloration in the TTC assay (supplemented with antimycin A) (**Figure 4A**) or the formation of a purple halo around colonies in the ferrozine assay (**Figure 4B**), *C. glabrata* showed no such activity. This was the case for our standard WT strain and also for a set of clinical strains isolated from different anatomical sites. Iron and copper starvation is known to increase surface reductase activity (Georgatsou and Alexandraki, 1999; Santos et al., 2003). Enhanced activity was indeed observed for the *C. albicans* WT with the TTC assay, but not using ferrozine (**Figures 4C,D** and Supplementary Figure 1). In contrast *C. glabrata* lacked any detectable surface ferric reductase activity even under these inducing conditions (Supplementary Figure 1). We continued to elucidate the function of the *C. glabrata* ferric reductases by creating Cg*fre6*Δ and Cg*fre8*Δ single and double deletion mutants; we were, however, not able to delete Cg*AIM14*. In accordance with the lack of surface ferric reductase activity and in contrast to mutants lacking other major components of the HA iron uptake system (**Figure 2**), *C. glabrata* ferric reductase mutants showed no defects in growth with ferritin, under moderate FeCl_3_ levels (**Figures 5A,B**) or under copper and iron starvation (Supplementary Figure 1). Thus, neither CgFre6 nor CgFre8 are required for HA iron uptake of *C. glabrata*.

Taken together, these results point to major differences between the surface ferric reduction properties of *C. albicans* and *S. cerevisiae* in comparison to *C. glabrata*. The absence of measurable ferric reductase activity in *C. glabrata* argues against surface reductase functions of CgFre6 or CgFre8, and indicates a loss of surface ferric reduction ability in *C. glabrata*.

### *C. glabrata*, *C. albicans*, and *S. cerevisiae* Exhibit Extracellular Ferric Reduction

**FIGURE 6:**
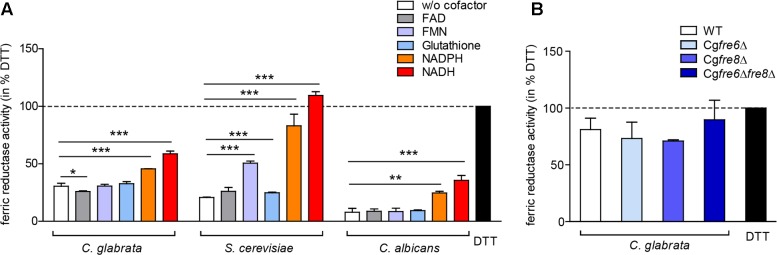
Extracellular ferric reduction in the culture supernatants of *C. glabrata*, *C. albicans*, and *S. cerevisiae*. oN cultures (in SD BR, pH 5.8) cells were harvested, the wet cell volume was determined and a cell-free culture supernatant was obtained via filtration. Extracellular ferric reduction in the supernatant was measured continuously for 3 h after addition of the detection mix (containing ferrozine, and ferric ammonium citrate as a ferric iron source) by spectrophotometrical detection of a ferrozine-Fe^2+^-complex. Data is shown for the 2 h time point after subtraction of the media control, as percentage of the positive control (the reductant DTT). Data is shown in biological triplicates as means ± standard deviation, for statistical analysis unpaired Student’s *t*-test was performed (^∗^*P* < 0.05, ^∗∗^*P* < 0.01, or ^∗∗∗^*P* < 0.001). **(A)** Extracellular ferric reduction activity of *C. glabrata*, *S. cerevisiae*, and *C. albicans* WT strains after addition of different cofactors with a final concentration of 250 μg/ml NADH, 250 μg/ml NADPH, 162.5 μM glutathione, 0.5 μM FMN, or 0.5 μM FAD. **(B)** Extracellular ferric reduction activity of *C. glabrata* WT, Cg*fre6*Δ, Cg*fre8*Δ and Cg*fre6*Δ*fre8*Δ with NADH as cofactor.

The importance of surface ferric reduction has been demonstrated in various bacteria and fungi (Schröder et al., 2003). However, some fungi such as *Histoplasma capsulatum* are also able to secrete either ferric reductases (Zarnowski and Woods, 2005; Zarnowski et al., 2008) or low molecular weight ferric reductants into the environment (Timmerman and Woods, 1999). We therefore postulated that *C. glabrata* might engage in a similar strategy to compensate for its lack of surface ferric reductase activity. To measure extracellular ferric reduction activity in culture supernatants of *C. glabrata*, *C. albicans*, and *S. cerevisiae*, we monitored conversion of Fe^3+^ to Fe^2+^ by spectrophotometric detection of Fe^2+^-ferrozine complexes, with the reductant 1,4-Dithiothreitol (DTT) as a positive control. For all analyzed species, ferric reduction activities were detected (**Figure 6A**).

Microbial ferric reductases typically act as oxido-reductases and require a cofactor as electron donor. This cofactor is frequently NAD(P)H (Schröder et al., 2003), and more rarely glutathione (Zarnowski and Woods, 2005; Zarnowski et al., 2008). In addition, certain bacteria additionally require flavins (FAD and FMN) as intermediate electron acceptor (Schröder et al., 2003). Accordingly, we tested the potential cofactor function of glutathione, FAD, FMN, NADH, or NADPH in extracellular ferric reduction (**Figure 6A**). Only addition of NADH or NADPH led to a significant increase in ferric reduction in all species. A slight increase upon FMN addition was additionally detected in *S. cerevisiae* supernatants. NADH was therefore chosen as a cofactor for further experiments.

Our data thus show that all three species are able to reduce ferric iron extracellularly.

### Extracellular Ferric Reduction Is Independent of Fre6 and Fre8 and Mediated by a Low-Molecular Weight Molecule

To test whether the putative *C. glabrata* ferric reductases contribute to extracellular ferric reduction, we analyzed culture supernatants of the Cg*FRE6* and Cg*FRE8* single and double deletion mutants in presence of NADH. The deletion mutants showed similar extracellular ferric reduction capabilities as the WT (**Figure 6B**), indicating that CgFre6 and CgFre8 are not only dispensable for surface activity, but also for extracellular ferric reduction by *C. glabrata*.

**FIGURE 7:**
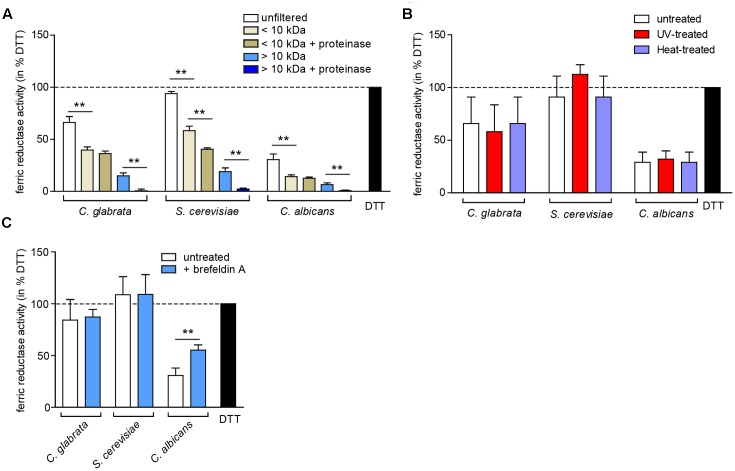
The extracellular ferric reduction activity is mediated by a low-molecular non-protein in *C. glabrata*, *C. albicans*, and *S. cerevisiae*. oN cultures (in SD BR, pH 5.8) cells were harvested, the wet cell volume was determined and a cell-free culture supernatant was obtained via filtration. Extracellular ferric reduction in the supernatant was measured continuously for 3 h after addition of the detection mix (containing NADH, ferrozine and ferric ammonium citrate) by spectrophotometrical detection of a ferrozine-Fe^2+^-complex. Data is shown for the 2 h time point after subtraction of the media control, as percentage of the positive control (the reductant DTT). Data is shown in biological triplicates as means ± standard deviation, for statistical analysis unpaired Student’s *t*-test was performed (^∗^*P* < 0.05, ^∗∗^*P* < 0.01, ^∗∗∗^*P* < 0.001). **(A)** Extracellular ferric reduction activity of *C. glabrata*, *S. cerevisiae*, and *C. albicans* WT strains after molecular weight fractioning of the culture supernatants. Analysis has been performed for the high molecular weight fraction (>10 kDa) and the low molecular weight fraction (<10 kDa) with or without addition of proteinase K (100 μg/ml, 30 min, 37°C). **(B)** Extracellular ferric reduction activity of *C. glabrata*, *S. cerevisiae*, and *C. albicans* WT strains after UV-treatment (120 mJ, 360 s) or heat-treatment (70°C, 20 min). **(C)** Extracellular ferric reduction activity of *C. glabrata*, *S. cerevisiae*, and *C. albicans* WT strains after addition of the protein secretion inhibitor brefeldin A (10 μg/ml) prior to oN culturing.

To elucidate whether the observed extracellular ferric reduction is dependent on another protein or a smaller molecule, we separated the supernatant into a low molecular weight fraction (<10 kDa) and a high molecular weight fraction (>10 kDa), and optionally treated both with proteinase K (**Figure 7A**). The majority of extracellular ferric reduction activity was found in the low molecular weight fraction for all species. As this activity was not (*C. glabrata* and *C. albicans*) or only slightly (*S. cerevisiae*) affected by proteinase K treatment, these data point to a small non-protein molecule as the main reducing agent. The high molecular weight fraction contributed only a very small reductase activity in all species, which was almost completely abolished by addition of proteinase K. This points to a minor contribution of a potentially secreted protein. In support of these findings, extracellular ferric reduction was not affected by heat or ultraviolet light (UV) treatment to denature proteins (**Figure 7B**), or by growth in presence of brefeldin A, an inhibitor of protein secretion in eukaryotes (Kossaczka et al., 1995) (**Figure 7C**).

In summary, major extracellular ferric reduction activity in the three analyzed species is likely carried out by a low molecular weight non-protein.

## Discussion

Exploitation of host iron sources and efficient iron uptake are essential for pathogens to survive and grow in the host. Our experiments confirmed again that *C. albicans* is able to use a broad spectrum of host iron sources including ferritin (Almeida et al., 2008), transferrin (Knight et al., 2005), hemin (Santos et al., 2003), and hemoglobin (Kuznets et al., 2014). The latter three served as iron sources preferably at alkaline pH, a condition also found in blood, where these iron sources are predominantly encountered by the fungus. Accordingly, our species-comparative analysis of iron acquisition systems detected the known ferritin and heme uptake receptors in *C. albicans*, while both, *S. cerevisiae* and *C. glabrata* seem to lack the necessary genes to exploit these iron sources. We cannot exclude that the current knowledge of iron uptake-related genes is not complete and that there are yet unknown host iron source-specific utilization systems in *C. glabrata* or *S. cerevisiae*. However, the fact that both species are unable to grow with transferrin, hemoglobin, or hemin as sole iron source seems to argue against such additional systems. It has been shown previously that hemin is not a suitable iron source for *C. glabrata* (Nevitt and Thiele, 2011), although earlier studies had reported hemolytic activity by *C. glabrata in vitro* (Luo et al., 2004; Malcok et al., 2009). This study adds transferrin as another potential iron source which seems inaccessible to *C. glabrata*.

The inability of *S. cerevisiae* to use all these host molecules may be considered not too surprising, as this yeast leads a generally non-pathogenic lifestyle. It seems more puzzling why *C. glabrata* is also not able to exploit transferrin, hemoglobin, or heme: While it underlines its close evolutionary relationship to the baker’s yeast, it raises the question of how this fungus is able to grow in the host environment and cause disease. In fact, the lack of the ability to use iron sources of the blood and the iron-related growth impairment of *C. glabrata* at alkaline pH suggests that *C. glabrata* is not well-adapted to typical alkaline host niches. Our data rather implies that *C. glabrata* depends on a restricted spectrum of host iron sources, especially compared to *C. albicans.* As non-bound iron is virtually non-existent in the host, these sources seem limited to ferritin and xenosiderophores, produced by other members of the microbial flora (Nevitt and Thiele, 2011).

Which system allows *C. glabrata* the exploitation of ferritin and the use of non-protein-bound iron? Our *in silico* analyses show the existence of a low-affinity (LA) iron uptake, a xenosiderophore uptake, and a HA iron uptake system. In previous studies, the disruption of LA iron uptake (Fet4) resulted in no apparent iron-related phenotype (Srivastava et al., 2014; Gerwien et al., 2016), suggesting a minor role for this system, similar maybe to *S. cerevisiae* (Dix et al., 1994). Iron acquisition mediated by uptake of siderophores produced by other species might play a role during interspecies commensal growth, e.g., in the gut, and it may also enhance resistance to the microbicidal activity of phagocytes (Haas et al., 2008; Nevitt and Thiele, 2011). In agreement with previous studies (Srivastava et al., 2014; Gerwien et al., 2016; Sharma et al., 2016), we found that ferritin and non-protein-bound iron uptake in *C. glabrata* depends on the third pathway: the reductive HA iron uptake system. However, for ferritin-mediated iron acquisition the detailed mechanism, including the receptors for binding of ferritin and the mode of iron release, remains to be elucidated in *C. glabrata*.

Ferric reduction is a critical initial step for the reductive HA iron uptake and is usually highly conserved in bacteria and fungi (Schröder et al., 2003). Surprisingly, in contrast to the large gene families in other pathogenic fungi (Dancis et al., 1992; Martins et al., 1998; Yun et al., 2001; Blatzer et al., 2011; Jeeves et al., 2011; Saikia et al., 2014; Xu et al., 2014), we identified in our *in silico* analysis only three putative ferric reductase orthologs in *C. glabrata*, none of them orthologous to known surface reductases in *C. albicans* or *S. cerevisiae*. Although we cannot formally exclude the presence of other ferric reductases with alternate domains, we were not able to find more proteins with a ferric reductase transmembrane component-like domain in the predicted *C. glabrata* protein sequences. In line with this, our assays showed, in contrast to *C. albicans* and *S. cerevisiae*, no surface ferric reduction ability of *C. glabrata in vitro*. However, we still observed iron-dependent expression for two of the three putative ferric reductase genes, Cg*FRE6* and Cg*FRE8*, although deletion of both genes resulted in no apparent iron-related phenotypes. While we failed to delete Cg*AIM14*, the lack of surface reductase activity of the WT as well as the lack of a signal peptide in CgAim14 makes it similarly highly unlikely that it plays a role in surface-based iron acquisition processes.

Our observations for Cg*FRE6* complement other studies, where a Cg*FRE6* deletion did not affect iron-dependent growth or intracellular iron accumulation (Srivastava et al., 2014). Deletion also affected virulence of *C. glabrata* only subtly, with a slightly reduced fungal burden in murine kidneys (Srivastava et al., 2014) and a small increase in survival of a *Drosophila melanogaster* model (Brunke et al., 2015). Overall, our data thus resemble findings in *S. cerevisiae*, as Sc*FRE6* displays a moderate iron-dependent expression (Shakoury-Elizeh et al., 2004), and its deletion resulted in no apparent phenotype under iron limitation (Ahmed Khan et al., 2000). Importantly, ScFre6 is localized at the vacuole (Huh et al., 2003) and not at the cell surface. It seems thus likely that Fre6 in *C. glabrata* has a similar localization and possibly a similar, thus-far unknown biological function as ScFre6.

In contrast, our observations for CgFre8 (having an iron-dependent gene expression and being dispensable for growth) do not match to what is known for ScFre8. A strain lacking Sc*FRE8* was unable to grow in low iron and was respiration-deficient (De Freitas et al., 2004), but expression of Sc*FRE8* was unaffected by changing iron levels (Georgatsou and Alexandraki, 1999). These differences indicate potentially distinct roles for Fre8 in both species, although Cg*FRE8* deletion was accompanied by lower survival rates in macrophages and diminished intracellular replication (Seider et al., 2014).

In conclusion, we cannot exclude a more subtle biological function of CgFre6 and CgFre8 that depends on additional factors besides iron availability. With these two reductases being dispensable for growth, our data indicates a general loss of surface-associated ferric reduction, which makes *C. glabrata* – to our knowledge – the first pathogenic fungus to lack this capability.

Consequently, we analyzed whether secreted ferric reductants might be present in the fungus to compensate for the lack of surface ferric reduction. Surprisingly, we found ferric reduction capacity in the supernatants of all three investigated species, and we wondered whether this process was dependent on an enzymatic process. Although excreted ferric reductases have been described previously in *H. capsulatum*, those are glutathione-dependent (Zarnowski and Woods, 2005; Zarnowski et al., 2008), whereas our data point toward an NAD(P)H-dependent process. In *C. albicans* it has been suggested that the ferric reductase CaCfl2 is secreted (Sorgo et al., 2011), but no secreted ferric reductases have been described in *C. glabrata* (or *S. cerevisiae*), and both species lack direct CaCfl2 orthologs. Furthermore, although CaCfl2 might contribute to *C. albicans* extracellular ferric reduction, we observed the majority of extracellular ferric reduction in the low-molecular weight fraction in all three species, and protein degradation as well as inhibition of the protein secretory pathway did not influence extracellular ferric reduction. Thus, our observations strongly indicate a secreted non-protein compound to be responsible for the extracellular ferric reduction activity in all three species.

In other fungi, many low-molecular compounds are known which can act as ferric reductants, such as 3-hydroxyanthranilate (3-HAA) and anthranilate in *S. cerevisiae* (Lesuisse et al., 1992), 3-HAA in *C. neoformans* (Nyhus et al., 1997; Jacobson et al., 1998; Jung et al., 2008), an unknown compound in *H. capsulatum* (Timmerman and Woods, 1999), terrein in *Aspergillus terreus* (Gressler et al., 2015), 2,5-dimethylhydroquinone in the brown-rot fungi such as *Gloeophyllum trabeum* (De Luca and Wood, 2000; Arantes and Milagres, 2008), as well as secreted oxalic and citric acids in other wood-decaying fungi (Arantes and Milagres, 2008) and possibly in *A. niger* (Johnson, 2003). However, in most of these species surface-bound ferric reductases are still responsible for the vast majority of iron reduction, and these compounds play only secondary roles. This applies also to *C. albicans* and *S. cerevisiae* with their broad array of surface ferric reductases. For these fungi, secreted reductants may benefit the overall reductive capacity, but are not strictly necessary. For *C. glabrata*, however, the lack of surface ferric reductase activity combined with its limited use of host iron sources makes this ability likely much more important for sustained growth and survival in the host. The nature of *C. glabrata*’s reductants is still unknown, but 3-HAA [as in *S. cerevisiae* (Lesuisse et al., 1992)] seems unlikely, as it is a metabolite of the *de novo* NAD-biosynthetic pathway (Li and Bao, 2007), which, in contrast to *S. cerevisiae* and *C. albicans* is not existent in *C. glabrata* (Kucharczyk et al., 1998; Li and Bao, 2007; Ma et al., 2007). Whether a pathway from host-derived NADH toward 3-HAA could exist is highly speculative, and currently seems a remote possibility at best. The second extracellular ferric reductant described for *S. cerevisiae*, anthranilate (Lesuisse et al., 1992), is a precursor of tryptophan biosynthesis in fungi (Braus, 1991) and might be a reasonable candidate responsible for the extracellular ferric reduction observed in *C. glabrata*.

The reason for the promoting effect of NAD(P)H on extracellular ferric reduction in *C. glabrata* and *C. albicans* (this study) as well as *S. cerevisiae* [this study and (Lesuisse et al., 1992)] still remains to be elucidated. NAD(P)H is a potential cofactor for oxido-reductases, however, our data indicate that non-enzymatic processes are mainly responsible for extracellular ferric reduction. In some fungi anthranilate can react with NAD(P)H in an enzyme-dependent process, producing the iron-chelating compound 2,3-dihydroxybenzoate (Anderson and Dagley, 1981; Kamath and Vaidyanathan, 1990; Chang et al., 2003), which is also a precursor of the iron siderophore enterobactin in *Salmonella enterica* and *Escherichia coli* (Graziano et al., 1974; Raymond et al., 2003). Anthranilate production by *C. glabrata* is likely (see above), however, the enzyme-independent nature of extracellular reduction speaks against a connection between anthranilate and NAD(P)H in *C. glabrata*.

Taken together, we propose here a novel iron acquisition strategy for *C. glabrata*, which differs from *C. albicans* and other pathogenic fungi. This strategy is characterized by the dependence on a restricted host iron source spectrum (ferritin and non-protein-bound iron) and a lack of surface ferric reduction activity while still relying on the HA iron uptake system. Instead *C. glabrata* possibly employs extracellular ferric reduction, mediated by a non-protein ferric reductant of low molecular weight.

## Materials and Methods

### Strains

The *C. glabrata* deletion mutant Cg*fre6*Δ was generated in an ATCC 2001 background by replacing the Cg*FRE6* with a *TEF1* promoter-driven nourseothricin resistance (*NAT1*) cassette (Schwarzmüller et al., 2014). The cassette was amplified with 500–1,000 bp gene-specific flanks by PCR (primer: Fre6 na fwd × Fre6 na rev) from a previously existing mutant (Schwarzmüller et al., 2014). The PCR-amplified insert (primer Fre6 fwd × Fre6 rev) was used for *C. glabrata* transformation using a modified heat shock method (Sanglard et al., 1996) with 45°C heat shock for 15 min. For the double mutant Cg*fre6*Δ*fre8*Δ, Cg*FRE6* was deleted in an ATCC 2001 *his3*Δ background strain (Schwarzmüller et al., 2014) as described above, followed by Cg*FRE8* deletion using Cg*HIS3* as an auxotrophy selection marker (including 861 bp promoter sequence and 232 bp terminator sequence): A *HIS3* fragment (primer HIS3 gene fwd × HIS3 gene rev) was fused with 1,000 bp *FRE8*-specific 5′- and 3′-flanks (primer Fre8-fwd-pUC19 × Fre8-HIS3 5′flank-BC, Fre8-HIS3 3′flank-BC × Fre8-rev-pUC19) and integrated into a *Xba*I-linearized pUC19 vector using the Infusion HD Cloning Kit (Clontech). The PCR-amplified insert (primer Fre8 fwd × Fre8 rev) was used for transformation. The transformants were plated onto YPD agar (2% agar, 1% yeast extract, 2% peptone, 2% glucose) containing 250 μg/ml nourseothricin, or SD agar supplemented with 0.079% complete supplement mix (CSM) without histidine (Formedium), 2.5 μM Cu_2_O, 2.5 μM CuSO_4_, 25 μM FeCl_3_, and 25 μM FeSO_4_. Knockout strains were verified by PCR (primer gene naCoP1 × gene naCoP4) and Sanger sequencing. Strains used in this study are shown in **Table 2** and all primers are listed in Supplementary Table 3.

**Table 2:** Strains used in this study.

Strain name	Description	Reference
***C. glabrata***		
WT	*C. glabrata* WT strain ATCC 2001	American type culture collection
Cg*fre6*Δ	ATCC 2001, *CAGL0C03333g*Δ::*NAT1*	This study
Cg*fre8*Δ	ATCC 2001, *CAGL0M07942g*Δ::*NAT1*	Seider et al., 2014
Cg*fre6*Δ*fre8*Δ	ATCC 2001 *his3*Δ, *CAGL0C03333g*Δ::*NAT1, CAGL0M07942g*Δ::*HIS3*	This study
Cg*ftr1*Δ	ATCC 2001, *CAGL0I06743g*Δ::*NAT1*	Gerwien et al., 2016
Cg*fet3*Δ	ATCC 2001, *CAGL0F06413g*Δ::*NAT1*	Gerwien et al., 2016
Cg*aft1*Δ	ATCC 2001, *CAGL0H03487g*Δ::*NAT1*	Gerwien et al., 2016
Cg*cth2*Δ	ATCC 2001, *CAGL0E01243g*Δ::*NAT1*	Gerwien et al., 2016
PEU597	Clinical Isolate from urine/catheter	Oliver Bader^#^
PEU598	Clinical Isolate from feces	Oliver Bader^#^
BAK602	Clinical Isolate from bronchio-alveolar lavage	Oliver Bader^#^
BAK616	Clinical Isolate from oral swab	Oliver Bader^#^
BAK617	Clinical Isolate from bronchial secretions	Oliver Bader^#^
BAK618	Clinical Isolate from vaginal swab	Oliver Bader^#^
BAK637	Clinical Isolate from midstream urine	Oliver Bader^#^
***S. cerevisiae***		
WT	*S. cerevisiae* WT strain ATCC9763	American type culture collection
***C. albicans***		
WT	*C. albicans* WT strain SC5314	Fonzi and Irwin, 1993

*Wild-type (WT), ^#^Human clinical isolates were a generous gift from Oliver Bader (Göttingen, Germany)*.

### Growth Curves

Strains were cultivated over-night (oN) at 37°C (*C. glabrata*) or 30°C (*C. albicans* and *S. cerevisiae*) in liquid synthetic defined media (SD: 0.67% YNB, 2% Glucose, 0.079% CSM [Formedium]) with 180°rpm shaking. They were then transferred to citrate-buffered SD (pH 5.8) containing 500 μM (for *C. albicans*) or 200 μM (for *C. glabrata* and *S. cerevisiae*) of the extracellular iron chelator bathophenanthrolinedisulfonic acid (BPS) to induce iron starvation. BPS precultures were incubated for 22 h, washed three times by centrifugation and re-elution in iron-free water, and then resuspended in iron-free water to on OD_600_ of 0.1. The yeast suspension (20 μl) was added to 180 μl citrate-buffered SD (pH 4.5, 5.8, or 7.3) containing 200 μM BPS and supplemented with either 100 μM FeCl_3_ (stock in 1% HCl), 100 μg/ml horse ferritin (stock in iron-free 5 mM HEPES, 0.15 M NaCl, 4× filtered through 50 KDa molecular weight cut-off columns [Amicon Ultra 0.5 ml]), 100 μg/ml transferrin (Calbiochem, stock in 0.15 M iron-free Na_2_CO_3_, 2× filtered through 50 KDa columns [Amicon Ultra 0.5 ml]), 0.1 mg/ml bovine hemoglobin (Sigma, stock in H_2_O), or 1 μM hemin (Sigma, stock in DMSO). Growth was recorded over 2 days in biological triplicates by OD_600_ measurement every 30 min (with intermittent shaking) at 37°C in a Tecan Infinite 200 ELISA reader.

### TTC-Based Surface Reductase Activity Assay

SD oN cultures were washed twice with iron-free water, and adjusted to 2 × 10^7^ cells/ml in iron-free water for spotting of 1:10 serial dilutions on unbuffered SD agar containing the metal chelator ethylenediaminetetraacetic acid – (EDTA) (3 μM), the iron chelator BPS (10 μM), or the copper chelator ammonium tetrathiomolybdate – (ATTM) (7 μM). For more alkaline conditions, SD agar was phosphate-buffered to pH 6.4. Plates were incubated oN at 37°C. For the negative control, one plate was inactivated after oN cultivation by heat treatment (1 h, 72°C). Subsequently, 1.5% agarose was melted in 1 × TAE and supplemented with 0.1% TTC (Roth), plus 20 μg/ml antimycin A (Sigma) to inhibit mitochondrial reduction activity (Hsu et al., 2011) where indicated. The plates were overlaid with this TTC agarose, and formation of red formazan, indicating cell surface reduction, was detected in biological triplicates after 1 h incubation.

### Ferrozine-Based Surface Ferric Reductase Activity Assay

Serial dilution growth plates were prepared as described before, but with an overlay mixture of 0.5% agarose, 10 mM MgCl_2_, 0.1 mg/ml NADH, 1 mg/ml ferrozine, 5 mg/ml ferric ammonium citrate instead. Formation of a purple halo around colonies, indicating surface ferric reductase activity, was documented in biological triplicates after 5 min incubation.

### Ferric Reductases Assay in Culture Supernatants

Ferric reduction activity was measured for all three species from stationary oN cultures. Preliminary experiments revealed that low pH medium [such as unbuffered rich medium (YPD) and minimal medium (SD)] or a low pH due to secretion of fungal metabolic products lead to increased background signals (not shown). We therefore chose buffered nutrient-limited medium for our assays, which resulted in the lowest background noise and satisfactory growth for all tested species – SD BR medium [0.67% YNB, 2% Glucose, 50% Britton Robinson buffer (40 mM H_3_BO_4_, 40 mM Acetic acid, 40 mM H_3_PO_4_, 3 g/L KCl, pH 5.8)]. Precultures were prepared in SD BR, and cultures were treated with an inhibitor of Golgi/ER-dependent protein secretion, brefeldin A (10 μg/ml) (Kossaczka et al., 1995) where indicated. The average wet cell volume per 200 μl oN culture was determined by cell volume tubes (TPP) for normalization. The cell-free supernatant was obtained by centrifugation for 4 min at 4,000 ×*g* and filtering through Minisart syringe filters (Sigma, 0.2 μm). Supernatant and media control were treated identically: Heat-treatment (70°C, 20 min), UV-treatment (120 mJ, 360 s), fractionation on molecular weight (Ultra 0.5 ml Amicon centrifugal protein filters, 10 kDa molecular weight cut-off with restoration of original volume of the high molecular weight fraction in adequate medium), or proteinase K-treatment (100 μg/ml, 30 min, 37°C). Culture supernatant or control (180 μl) were combined with 20 μl detection mix consisting of five volumes Britton-Robinson buffer, 1 volume 1 M MgCl_2_, 0.5 volumes 15 mg/ml ammonium ferric citrate, 1 volume 20 mM ferrozine, and 2.5 volumes of potential cofactor (stock either 10 mg/ml NADH, 10 mg/ml NADPH, 6.5 mM glutathione, 20 μM FMN, or 20 μM FAD). As positive control, 5 mM of the reductant DTT was added. Iron reduction (as ferrous iron binding to ferrozine) was detected by OD_568_ measurement every 5 min for 4 h at 37°C in a Tecan Infinite 200 ELISA reader in biological triplicates. Data of the 2 h time point was normalized to the initial wet cell volume and adjusted to a scale from 0% (medium control) to 100% (DTT).

### Sample Preparation for RNA Isolation

Sample preparation and RNA isolation was conducted as described previously (Gerwien et al., 2016). Briefly, an SD oN culture was harvested, washed, and 1 × 10^7^ cells/ml were inoculated into iron-free citrate-buffered SD (pH 5.8) supplemented with 5 μM FeCl_3_ and grown for 4 h at 37°C and 180 rpm. The 0 h sample was immediately frozen in liquid nitrogen and stored at -80°C. The remaining culture was washed four times with iron-free SD (start time set as first contact with the medium) and incubated in the same medium for the 1 and 4 h samples. RNA was isolated with the RNAeasy Mini Kit (Qiagen), and quality was verified using the Agilent 2100 Bioanalyzer Nanochip system according to the manufacturer’s protocol. The concentration was determined using a NanoDrop 1000 instrument.

### Quantitative Real-Time PCR (qRT-PCR)

Quantitative real-time PCR was conducted as described previously (Gerwien et al., 2016). Briefly, 600 ng high-quality RNA was treated with DNAse (Epicentre) and reversely transcribed into cDNA using oligo-dT primers and Superscript III (Invitrogen). 1 μl diluted cDNA (1:20) was used for gene expression analysis in a C1000 thermocycler (Bio-Rad, CFX96 Realtime system) with the EvaGreen system (Bio & Sell). The expression rates were determined in biological triplicates and normalized to the house keeping genes *EFB1* and *EFT2* using Bio-Rad CFX Manager 3.1.

### *In Silico* Analysis, Databases and Statistics

Information about gene orthologs, protein structure, and BLAST results were obtained from the *Candida* Genome Database (CGD)^1^ and the *Saccharomyces* Genome Database (SGD)^2^. BLAST search for proteins with a predicted ferric reductase domain in *C. glabrata* (pfam family PF01794, ferric reductase transmembrane component-like domain) was performed using the domain sequence of ScFre1 and CaFre10 as query. Additionally, the presence of additional conserved protein domains were predicted by CGD and SGD-associated prediction tools (SignalP, TMHMM, Pfam) and verified by SMART^3^. Subcellular localization was predicted by TargetP 1.1^4^. GraphPad Prism 5 (GraphPad Inc.) was used for statistics. All data are reported as the mean ± SEM or standard deviation where appropriate, and two-tailed, unpaired Student’s *t*-test was performed, if not stated otherwise. Statistically significant results were marked as ^∗^*P* < 0.05, ^∗∗^*P* < 0.01, ^∗∗∗^*P* < 0.001.

## Author Contributions

The authors FG, LK, and BH designed the study. FG, AS, and SW performed and the experiments and acquired the data. FG, AS, and LK evaluated and interpreted the results. FG designed the figures and FG, SB, LK, and BH wrote and revised the manuscript.

## Conflict of Interest Statement

The authors declare that the research was conducted in the absence of any commercial or financial relationships that could be construed as a potential conflict of interest.

## Footnotes

1 www.candidagenome.org

2 www.yeastgenome.org

3 smart.embl-heidelberg.de

4 www.cbs.dtu.dk/services/TargetP/
