# Supplementary material for: The Fungal Pathogen Candida glabrata Does Not Depend on Surface Ferric Reductases for Iron Acquisition
Source: Front Microbiol. 2017 Jun 8;8:1055. doi: 10.3389/fmicb.2017.01055 (PMC5463049; doi:10.3389/fmicb.2017.01055)
Supplement: Supplementary file 1 [file Table_1.DOCX]

Supplementary Table 1. Intracellular iron homeostasis in *C. glabrata*, *S. cerevisiae* and *C. albicans*

| Process | Function | *C. glabrata* | *S. cerevisiae* | *C. albicans* |
| --- | --- | --- | --- | --- |
| Vacuolar iron transport | Iron importer | Ccc1 (Gerwien et al., 2016) | Ccc1 (Li et al., 2001) | Ccc1^a)^ (Xu et al., 2014) |
|  | LA-metal transporter | Smf1^#^ | Smf1^b)^ (Chen et al., 1999;Portnoy et al., 2002) | Smf1^#^ |
|  | Metal exporter | Smf3^#^ | Smf3 (Cohen et al., 2000;Portnoy et al., 2002) | Smf3 (Xu et al., 2014) |
|  | Metal exporter complex | Fth1/unknown (Gerwien et al., 2016) | Fth1/Fet5 (Urbanowski and Piper, 1999) | Fth1 or Fth2/unknown (Lan et al., 2004;Chen et al., 2011) |
| Mitochondrial iron transport | Frataxin | Yfh1 (Srivastava et al., 2014;Gerwien et al., 2016) | Yfh1 (Park et al., 2003;Bulteau et al., 2004) | Yfh1 (Santos et al., 2004) |
|  | ABC exporter of Fe-S clusters | Atm1 (Gerwien et al., 2016) | Atm1 (Kispal et al., 1997;Kispal et al., 1999) | Atm1 (Lan et al., 2004) |
|  | Metal exporter | Mmt1^#^, Mmt2 (Gerwien et al., 2016) | Mmt1, Mmt2 (Li and Kaplan, 1997) | Mmt1^#^ |
|  | Metal importer | Mrs3^#^, Mrs4^#^ | Mrs3, Mrs4 (Muhlenhoff et al., 2003;Froschauer et al., 2009) | Mrs4 (Xu et al., 2014) |

| ATP binding cassette (ABC), Low affinity (LA), Iron-sulfur cluster (Fe-S cluster), # ortholog present but no iron related function has been reported. |
| --- |
| 1. CaCcc1 has also been described as localized to the hyphal tip (Elson et al., 2009). 2. ScSmf1has been shown to act predominantly as manganese transporter with a secondary function for ferrous iron transport. |

Bulteau, A.L., O'neill, H.A., Kennedy, M.C., Ikeda-Saito, M., Isaya, G., and Szweda, L.I. (2004). Frataxin acts as an iron chaperone protein to modulate mitochondrial aconitase activity. *Science* 305**,** 242-245.

Chen, C., Pande, K., French, S.D., Tuch, B.B., and Noble, S.M. (2011). An iron homeostasis regulatory circuit with reciprocal roles in *Candida albicans* commensalism and pathogenesis. *Cell Host Microbe* 10**,** 118-135.

Chen, X.Z., Peng, J.B., Cohen, A., Nelson, H., Nelson, N., and Hediger, M.A. (1999). Yeast *SMF1* mediates H(+)-coupled iron uptake with concomitant uncoupled cation currents. *J Biol Chem* 274**,** 35089-35094.

Cohen, A., Nelson, H., and Nelson, N. (2000). The family of *SMF* metal ion transporters in yeast cells. *J Biol Chem* 275**,** 33388-33394.

Elson, S.L., Noble, S.M., Solis, N.V., Filler, S.G., and Johnson, A.D. (2009). An RNA transport system in *Candida albicans* regulates hyphal morphology and invasive growth. *PLoS Genet* 5**,** e1000664.

Froschauer, E.M., Schweyen, R.J., and Wiesenberger, G. (2009). The yeast mitochondrial carrier proteins Mrs3p/Mrs4p mediate iron transport across the inner mitochondrial membrane. *Biochim Biophys Acta* 1788**,** 1044-1050.

Gerwien, F., Safyan, A., Wisgott, S., Hille, F., Kaemmer, P., Linde, J., Brunke, S., Kasper, L., and Hube, B. (2016). A Novel Hybrid Iron Regulation Network Combines Features from Pathogenic and Nonpathogenic Yeasts. *MBio* 7.

Kispal, G., Csere, P., Guiard, B., and Lill, R. (1997). The ABC transporter Atm1p is required for mitochondrial iron homeostasis. *FEBS Lett* 418**,** 346-350.

Kispal, G., Csere, P., Prohl, C., and Lill, R. (1999). The mitochondrial proteins Atm1p and Nfs1p are essential for biogenesis of cytosolic Fe/S proteins. *EMBO J* 18**,** 3981-3989.

Lan, C.Y., Rodarte, G., Murillo, L.A., Jones, T., Davis, R.W., Dungan, J., Newport, G., and Agabian, N. (2004). Regulatory networks affected by iron availability in *Candida albicans*. *Mol Microbiol* 53**,** 1451-1469.

Li, L., Chen, O.S., Mcvey Ward, D., and Kaplan, J. (2001). *CCC1* is a transporter that mediates vacuolar iron storage in yeast. *J Biol Chem* 276**,** 29515-29519.

Li, L., and Kaplan, J. (1997). Characterization of two homologous yeast genes that encode mitochondrial iron transporters. *J Biol Chem* 272**,** 28485-28493.

Muhlenhoff, U., Stadler, J.A., Richhardt, N., Seubert, A., Eickhorst, T., Schweyen, R.J., Lill, R., and Wiesenberger, G. (2003). A specific role of the yeast mitochondrial carriers MRS3/4p in mitochondrial iron acquisition under iron-limiting conditions. *J Biol Chem* 278**,** 40612-40620.

Park, S., Gakh, O., O'neill, H.A., Mangravita, A., Nichol, H., Ferreira, G.C., and Isaya, G. (2003). Yeast frataxin sequentially chaperones and stores iron by coupling protein assembly with iron oxidation. *J Biol Chem* 278**,** 31340-31351.

Portnoy, M.E., Jensen, L.T., and Culotta, V.C. (2002). The distinct methods by which manganese and iron regulate the Nramp transporters in yeast. *Biochem J* 362**,** 119-124.

Santos, R., Buisson, N., Knight, S.A., Dancis, A., Camadro, J.M., and Lesuisse, E. (2004). *Candida albicans* lacking the frataxin homologue: a relevant yeast model for studying the role of frataxin. *Mol Microbiol* 54**,** 507-519.

Srivastava, V.K., Suneetha, K.J., and Kaur, R. (2014). A systematic analysis reveals an essential role for high-affinity iron uptake system, haemolysin and CFEM domain-containing protein in iron homoeostasis and virulence in *Candida glabrata*. *Biochem J* 463**,** 103-114.

Urbanowski, J.L., and Piper, R.C. (1999). The iron transporter Fth1p forms a complex with the Fet5 iron oxidase and resides on the vacuolar membrane. *J Biol Chem* 274**,** 38061-38070.

Xu, N., Dong, Y., Cheng, X., Yu, Q., Qian, K., Mao, J., Jia, C., Ding, X., Zhang, B., Chen, Y., Zhang, B., Xing, L., and Li, M. (2014). Cellular iron homeostasis mediated by the Mrs4-Ccc1-Smf3 pathway is essential for mitochondrial function, morphogenesis and virulence in *Candida albicans*. *Biochim Biophys Acta* 1843**,** 629-639.
