# Supplementary material for: The Fungal Pathogen Candida glabrata Does Not Depend on Surface Ferric Reductases for Iron Acquisition
Source: Front Microbiol. 2017 Jun 8;8:1055. doi: 10.3389/fmicb.2017.01055 (PMC5463049; doi:10.3389/fmicb.2017.01055)
Supplement: Supplementary file 2 [file Table_2.DOCX]

Supplementary Table 2. Function of the postulated ferric reductases in *S. cerevisiae* and *C. albicans*

| Ferric reductase | Function |
| --- | --- |
| ScFre1 | Major surface reductase (Dancis et al., 1990;Anderson et al., 1992;Georgatsou and Alexandraki, 1994), Cupric reductase (Hassett and Kosman, 1995;Georgatsou et al., 1997;Georgatsou and Alexandraki, 1999), Siderophore reduction (Yun et al., 2001) |
| ScFre2 | Major surface reductase (Anderson et al., 1992;Georgatsou and Alexandraki, 1994), Cupric reductase (Hassett and Kosman, 1995;Georgatsou et al., 1997), Siderophore reduction (Yun et al., 2001) |
| ScFre3 | Ferric reductase (Martins et al., 1998;Georgatsou and Alexandraki, 1999), Siderophore reduction (Yun et al., 2001) surface localization (Yun et al., 2001) |
| ScFre4 | Ferric reductase (Martins et al., 1998;Georgatsou and Alexandraki, 1999), Siderophore reduction (Yun et al., 2001) |
| ScFre5 | Potential ferric reductase (Georgatsou and Alexandraki, 1999), Mitochondrial localization (Sickmann et al., 2003) |
| ScFre6 | Potential ferric reductase (Martins et al., 1998;Georgatsou and Alexandraki, 1999;Yun et al., 2001), Vacuolar localization (Huh et al., 2003) |
| ScFre7 | Cupric reductase (Martins et al., 1998;Georgatsou and Alexandraki, 1999) |
| ScFre8 | Ferric reductase (Georgatsou and Alexandraki, 1999;De Freitas et al., 2004) |
| ScAim14 | ER localization (Yofe et al., 2016) |
| CaCfl1 | Ferric reductase (Hammacott et al., 2000;Knight et al., 2005) |
| CaCfl2 | Ferric reductase (Lan et al., 2004), Alkaline induced (Bensen et al., 2004;Baek et al., 2008), Possibly secreted (Sorgo et al., 2011) |
| CaFre3 | Unknown |
| CaFre10 | Major surface reductase (Knight et al., 2002;Santos et al., 2003;Jeeves et al., 2011), Cupric reductase (Knight et al., 2002) |
| CaFre4 | Unknown |
| CaCfl4 | Potential ferric reductase (Lan et al., 2004;Singh et al., 2011) |
| CaCfl5 | Potential ferric reductase (Lan et al., 2004;Knight et al., 2005;Chen et al., 2011;Singh et al., 2011) |
| CaFre6 | Unknown |
| CaCfl11 | Unknown |
| CaFre9 | Ferric reductase (Knight et al., 2005;Chen et al., 2011;Singh et al., 2011), Alkaline induced (Bensen et al., 2004) |
| CaFre7 | Major surface ferric reductase (Jeeves et al., 2011), Cupric reductase (Woodacre et al., 2008;Jeeves et al., 2011) |
| CaC1_09780C | Unknown |
| CaAim14 | Unknown |
| CaFrp1 | Ferric reductase (Lan et al., 2004;Baek et al., 2008;Singh et al., 2011), Alkaline induced (Bensen et al., 2004;Liang et al., 2009) |
| CaFre30 | Unknown |
| CaCR_07290W | Alkaline induced (Bensen et al., 2004) |
| CaFrp2 | Ferric reductase (Chen et al., 2011), Alkaline induced (Bensen et al., 2004) |

Anderson, G.J., Lesuisse, E., Dancis, A., Roman, D.G., Labbe, P., and Klausner, R.D. (1992). Ferric iron reduction and iron assimilation in *Saccharomyces cerevisiae*. *J Inorg Biochem* 47**,** 249-255.

Baek, Y.U., Li, M., and Davis, D.A. (2008). *Candida albicans* ferric reductases are differentially regulated in response to distinct forms of iron limitation by the Rim101 and CBF transcription factors. *Eukaryot Cell* 7**,** 1168-1179.

Bensen, E.S., Martin, S.J., Li, M., Berman, J., and Davis, D.A. (2004). Transcriptional profiling in *Candida albicans* reveals new adaptive responses to extracellular pH and functions for Rim101p. *Mol Microbiol* 54**,** 1335-1351.

Chen, C., Pande, K., French, S.D., Tuch, B.B., and Noble, S.M. (2011). An iron homeostasis regulatory circuit with reciprocal roles in *Candida albicans* commensalism and pathogenesis. *Cell Host Microbe* 10**,** 118-135.

Dancis, A., Klausner, R.D., Hinnebusch, A.G., and Barriocanal, J.G. (1990). Genetic evidence that ferric reductase is required for iron uptake in *Saccharomyces cerevisiae*. *Mol Cell Biol* 10**,** 2294-2301.

De Freitas, J.M., Kim, J.H., Poynton, H., Su, T., Wintz, H., Fox, T., Holman, P., Loguinov, A., Keles, S., Van Der Laan, M., and Vulpe, C. (2004). Exploratory and confirmatory gene expression profiling of mac1Delta. *J Biol Chem* 279**,** 4450-4458.

Georgatsou, E., and Alexandraki, D. (1994). Two distinctly regulated genes are required for ferric reduction, the first step of iron uptake in *Saccharomyces cerevisiae*. *Mol Cell Biol* 14**,** 3065-3073.

Georgatsou, E., and Alexandraki, D. (1999). Regulated expression of the *Saccharomyces cerevisiae* Fre1p/Fre2p Fe/Cu reductase related genes. *Yeast* 15**,** 573-584.

Georgatsou, E., Mavrogiannis, L.A., Fragiadakis, G.S., and Alexandraki, D. (1997). The yeast Fre1p/Fre2p cupric reductases facilitate copper uptake and are regulated by the copper-modulated Mac1p activator. *J Biol Chem* 272**,** 13786-13792.

Hammacott, J.E., Williams, P.H., and Cashmore, A.M. (2000). *Candida albicans* *CFL1* encodes a functional ferric reductase activity that can rescue a *Saccharomyces cerevisiae fre1* mutant. *Microbiology* 146 ( Pt 4)**,** 869-876.

Hassett, R., and Kosman, D.J. (1995). Evidence for Cu(II) reduction as a component of copper uptake by *Saccharomyces cerevisiae*. *J Biol Chem* 270**,** 128-134.

Huh, W.K., Falvo, J.V., Gerke, L.C., Carroll, A.S., Howson, R.W., Weissman, J.S., and O'shea, E.K. (2003). Global analysis of protein localization in budding yeast. *Nature* 425**,** 686-691.

Jeeves, R.E., Mason, R.P., Woodacre, A., and Cashmore, A.M. (2011). Ferric reductase genes involved in high-affinity iron uptake are differentially regulated in yeast and hyphae of *Candida albicans*. *Yeast* 28**,** 629-644.

Knight, S.A., Lesuisse, E., Stearman, R., Klausner, R.D., and Dancis, A. (2002). Reductive iron uptake by *Candida albicans*: role of copper, iron and the TUP1 regulator. *Microbiology* 148**,** 29-40.

Knight, S.A., Vilaire, G., Lesuisse, E., and Dancis, A. (2005). Iron acquisition from transferrin by *Candida albicans* depends on the reductive pathway. *Infect Immun* 73**,** 5482-5492.

Lan, C.Y., Rodarte, G., Murillo, L.A., Jones, T., Davis, R.W., Dungan, J., Newport, G., and Agabian, N. (2004). Regulatory networks affected by iron availability in *Candida albicans*. *Mol Microbiol* 53**,** 1451-1469.

Liang, Y., Gui, L., Wei, D.S., Zheng, W., Xing, L.J., and Li, M.C. (2009). *Candida albicans* ferric reductase *FRP1* is regulated by direct interaction with Rim101p transcription factor. *FEMS Yeast Res* 9**,** 270-277.

Martins, L.J., Jensen, L.T., Simon, J.R., Keller, G.L., and Winge, D.R. (1998). Metalloregulation of FRE1 and FRE2 homologs in *Saccharomyces cerevisiae*. *J Biol Chem* 273**,** 23716-23721.

Santos, R., Buisson, N., Knight, S., Dancis, A., Camadro, J.M., and Lesuisse, E. (2003). Haemin uptake and use as an iron source by *Candida albicans*: role of CaHMX1-encoded haem oxygenase. *Microbiology* 149**,** 579-588.

Sickmann, A., Reinders, J., Wagner, Y., Joppich, C., Zahedi, R., Meyer, H.E., Schonfisch, B., Perschil, I., Chacinska, A., Guiard, B., Rehling, P., Pfanner, N., and Meisinger, C. (2003). The proteome of *Saccharomyces cerevisiae* mitochondria. *Proc Natl Acad Sci U S A* 100**,** 13207-13212.

Singh, R.P., Prasad, H.K., Sinha, I., Agarwal, N., and Natarajan, K. (2011). Cap2-HAP complex is a critical transcriptional regulator that has dual but contrasting roles in regulation of iron homeostasis in *Candida albicans*. *J Biol Chem* 286**,** 25154-25170.

Sorgo, A.G., Heilmann, C.J., Dekker, H.L., Bekker, M., Brul, S., De Koster, C.G., De Koning, L.J., and Klis, F.M. (2011). Effects of fluconazole on the secretome, the wall proteome, and wall integrity of the clinical fungus *Candida albicans*. *Eukaryot Cell* 10**,** 1071-1081.

Woodacre, A., Mason, R.P., Jeeves, R.E., and Cashmore, A.M. (2008). Copper-dependent transcriptional regulation by *Candida albicans* Mac1p. *Microbiology* 154**,** 1502-1512.

Yofe, I., Weill, U., Meurer, M., Chuartzman, S., Zalckvar, E., Goldman, O., Ben-Dor, S., Schutze, C., Wiedemann, N., Knop, M., Khmelinskii, A., and Schuldiner, M. (2016). One library to make them all: streamlining the creation of yeast libraries via a SWAp-Tag strategy. *Nat Methods* 13**,** 371-378.

Yun, C.W., Bauler, M., Moore, R.E., Klebba, P.E., and Philpott, C.C. (2001). The role of the *FRE* family of plasma membrane reductases in the uptake of siderophore-iron in *Saccharomyces cerevisiae*. *J Biol Chem* 276**,** 10218-10223.
