# Supplementary material for: The Fungal Pathogen Candida glabrata Does Not Depend on Surface Ferric Reductases for Iron Acquisition
Source: Front Microbiol. 2017 Jun 8;8:1055. doi: 10.3389/fmicb.2017.01055 (PMC5463049; doi:10.3389/fmicb.2017.01055)
Supplement: Supplementary file 3 [file Table_3.DOCX]

**Supplementary Table 3. Primers used in this study.**

| Application context | Name | Sequence (5‘ 🡪 3‘) |
| --- | --- | --- |
| General Primers |  |  |
|  | puC19 MCS fwd | GGCGATTAAGTTGGGTAACG |
|  | puC19 MCS rev | TGGAATTGTGAGCGGATAAC |
|  | NAT up rev | GAACCATCCAAAGCTTCAATAG |
|  | NAT dn fwd | TTGTGGTTGGAAGTTACCAATG |
|  | HIS3 up rev | GGCGATTTGATTGTTACGTTAC |
|  | HIS3 dn forw | ATAGAACACAGCCCACAGCTAC |
|  | U1 fwd | CCGCTGCTAGGCGCGCCGTG |
|  | D1 rev | GCAGGGATGCGGCCGCTGAC |
| Gene KO primers | Fre6 na fwd | TGCGACTCGCCTTCTTCCTCTC |
|  | Fre6 na rev | GCTGCTCTGCCACATCAGCTTC |
|  | Fre6 na CoP1 | GCCAAGGTAGTATGGTATACAC |
|  | Fre6 na CoP4 | CAGCTTGTTGTTCATTCGTTTG |
|  | HIS3 gene fwd | TGATACACGTATCCCAAGAATG |
|  | HIS3 gene rev | ATATCGTGTTCGTGCGTATTTC |
|  | Fre8-fwd-pUC19 | GCAGGTCGACTCTAGTAGACTTTGCGATCTAAACAGATCC |
|  | Fre8-rev-pUC19 | CCGGGGATCCTCTAGTAAGAGATTCTTGGCTTTTCACTTC |
|  | Fre8-HIS3 5'flank | GGGATACGTGTATCAGTCCAGAGATTTATGCAGCTGTATG |
|  | Fre8-HIS3 3'flank | GCACGAACACGATATGTACAACCCTTTCTATTGTGAGAAC |
|  | Fre8 na CoP1 | TGGATAATTGCCAGAGACGA |
|  | Fre8 na CoP4 | TTGGCAATTGAATGTTTAGAGC |
|  | Fre8-fwd | TAGACTTTGCGATCTAAACAGATCC |
|  | Fre8-rev | TAAGAGATTCTTGGCTTTTCACTTC |
| qRT-PCT primers |  |  |
|  | qRT FRE6 fwd | GAATCTTCCTCGATGCTTGTAG |
|  | qRT FRE6 rev | TCAGAATTGGCCATTCTTTCAG |
|  | qRT FRE8 fwd | TCACGGATGTGTTCAAGAATG |
|  | qRT FRE8 rev | CTCCATGCACCAGTTGTAGTC |
|  | qRT AIM14 fwd | TGAGGGTGAAAGGATGGATTG |
|  | qRT AIM14 rev | ACTTCGGAAGCAAAGTGGATAC |
|  | EFB1-RT-P1 | GACACTGTCAAGGAATTGAACAC |
|  | EFB1-RT-P2 | GAAGCAGCTGGGAAGGAGT |
|  | qRT EFT2 HK fwd | TACTCTAAACTCCGACCCATTG |
|  | qRT EFT2 HK rev | TCCTTCATACCGTGTCTCTTAC |

**Underlined: primer overhangs**

**Grey: homolog to *Xba*1 linearized vector pUC19**

**Green: homolog to *HIS3* flanking regions**
