# Supplementary figures and images for: The Fungal Pathogen Candida glabrata Does Not Depend on Surface Ferric Reductases for Iron Acquisition
Source: Front Microbiol. 2017 Jun 8;8:1055. doi: 10.3389/fmicb.2017.01055 (PMC5463049; doi:10.3389/fmicb.2017.01055)

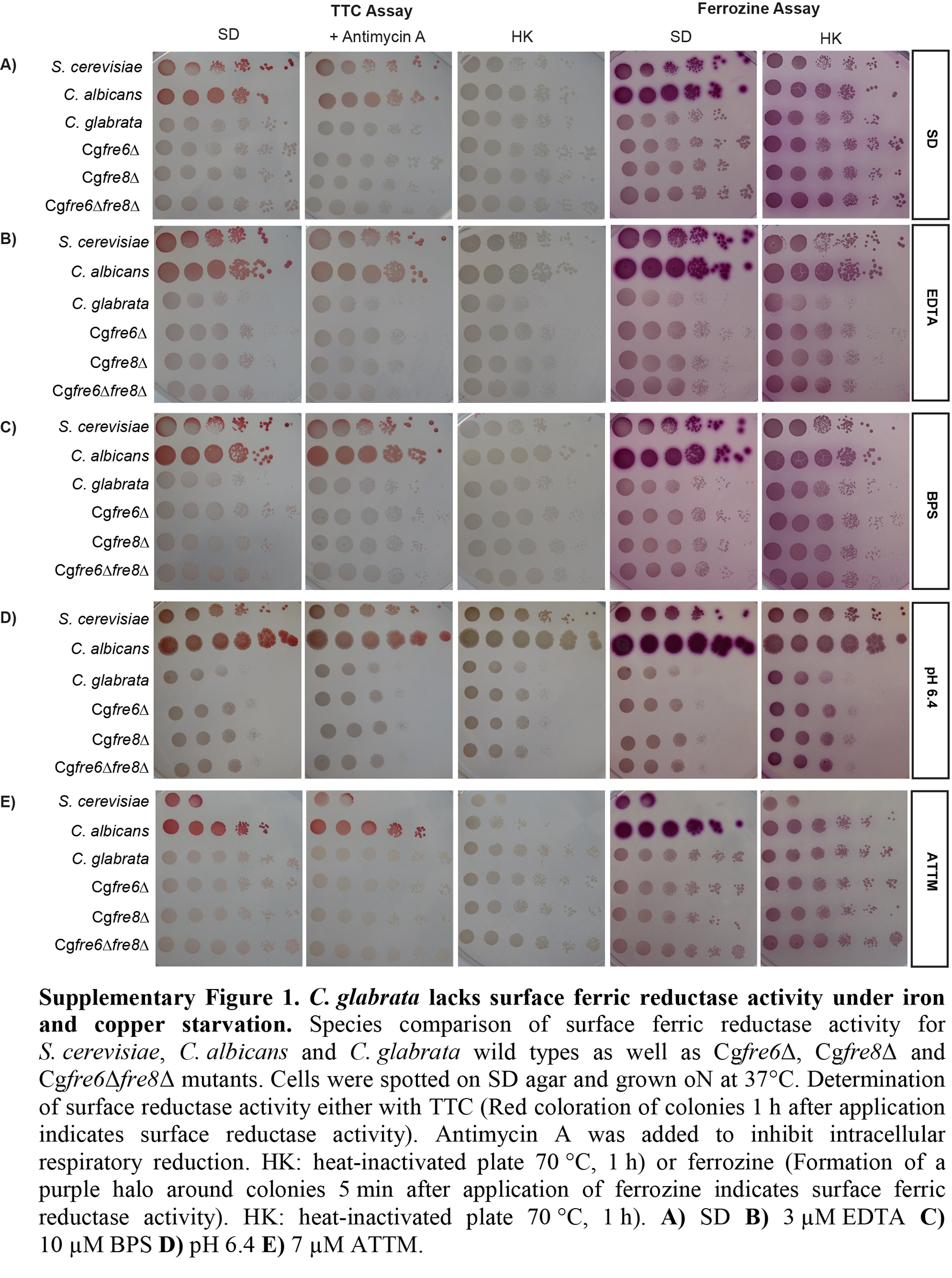

Supplement: Supplementary file 4 [file Image_1.TIF]
